# Refugial isolation and range expansions drive the genetic structure of *Oxyria sinensis* (Polygonaceae) in the Himalaya-Hengduan Mountains

**DOI:** 10.1038/srep10396

**Published:** 2015-05-27

**Authors:** Lihua Meng, Gang Chen, Zhonghu Li, Yongping Yang, Zhengkun Wang, Liuyang Wang

**Affiliations:** 1 School of Life Sciences, Engineering Research Center of Sustainable Development and Utilization of Biomass Energy Ministry of Education, Yunnan Normal University, Kunming 650092, Yunnan, P. R. China; 2 Graduate School of Oceanography, University of Rhode Island, 02881, RI USA; 3 Key Laboratory of Resource Biology and Biotechnology in Western China (Ministry of Education), College of Life Sciences, Northwest University, Xi’an 710069, Shanxi, P. R.China; 4 Kunming Institute of Botany, Chinese Academy of Sciences, Kunming 650204, Yunnan, P. R. China; 5 Department of Biology, Duke University, Durham, 27708, NC USA

## Abstract

The formation of the Mekong-Salween Divide and climatic oscillations in Pleistocene were the main drivers for the contemporary diversity and genetic structure of plants in the Himalaya-Hengduan Mountains (HHM). To identify the relative roles of the two historical events in shaping population history of plants in HHM, we investigated the phylogeographic pattern of *Oxyria sinensis*, a perennial plant endemic to the HHM. Sixteen chloroplast haplotypes were identified and were clustered into three phylogenetic clades. The age of the major clades was estimated to be in the Pleistocene, falling into several Pleistocene glacial stages and postdating the formation of the Mekong-Salween Divide. Range expansions occurred at least twice in the early and middle Pleistocene, but the spatial genetic distribution rarely changed since the Last Glacial Maximum. Our results suggest that temporary mountain glaciers may act as barriers in promoting the lineage divergence in *O. sinensis* and that subsequential range expansions and secondary contacts might reshape the genetic distribution in geography and blur the boundary of population differentiation created in the earlier glacial stages. This study demonstrates that Pleistocene climatic change and mountain glaciers, rather than the Mekong-Salween Divide, play the primary role in shaping the spatial genetic structure of *O. sinensis*.

Inferring the evolutionary causes that drive genetic diversity of a population over time and space has been one of the central topics in phylogeography^1^,^2^,^3^. Over the past two decades, DNA-based phylogeographic studies have greatly advanced our understanding of demographic dynamics and the evolutionary history of plants in response to historical events at a much finer time and spatial scale. These events include geological changes and climate changes, such as the formation of vicariance, the uplift of mountains, and climatic oscillations associated with Pleistocene glaciers. However, how geological and climate changes affect demographic processes, genetic differentiation, and even speciation remains poorly understood. It is of particular interest to study global biodiversity hotspot regions^4^, which feature exceptional concentrations of endemic species and habitat changes.

The Himalaya-Hengduan Mountains (HHM) have been recognized as one of the worldwide global biodiversity “hotspots”^4^, containing more than 12,000 plant species, more than 20% of which are endemic^4^,^5^,^6^,^7^,^8^. Incredibly high inter-/intra-specific diversification rates of plants have been documented in this region and hypothetically attributed to the geological uplifts of Qinghai-Tibetan Plateau (QTP) and/or recurrent Pleistocene glacial-interglacial climate oscillation^9^,^10^,^11^. However, the relative roles of these two historical forces in generating genetic diversity in this region are inconsistent among previous studies, leading to two contrasting hypotheses: a geological hypothesis emphasizing a major role for geological impacts and a climatic hypothesis highlighting the role of Pleistocene climate changes^10^,^12^,^13^.

According to the geological hypothesis, a continuous population might have been broken into spatially isolated subpopulations when new mountains formed during the uplifts of the QTP approximately 40 million years ago (Mya). Those isolated subpopulations eventually evolved into genetically distinct lineages via the accumulation of genetic differences and via adaptation to the local environment. This hypothesis has been strongly supported by the geological, ecological and recent genetic evidence. The HHM serve as the southeast boundary of the QTP and include numerous northwest-southeast high mountains with deeply incised valleys and rivers resulting from the extensive uplift movement of the QTP starting 40 Mya^14^. This region has an altitude range of ca. 1,000 to 6,000 m a.s.l., creating abundant ecological heterogeneity and diverse niches for plants^14^. Moreover, the geological changes have been episodic rather than steady. For example, the latest uplifts of the QTP between the late Miocene and Pliocene have greatly modified the tectonic morphology of the HHM, thereby creating novel geological disjunctions and rearranging the river drainage system^15^. Patterns of phylogeographic disjunctions that are concordant with the geological barriers have been found for a number of different plants^9^,^10^,^16^. A classic case was the Mekong-Salween Divide, a geological barrier that arose ca. 4 Mya and has been recognized for nearly a century^17^. The Mekong-Salween Divide was believed to have driven the population divergence of two plant species, *Sinopodophyllum hexandrum*^10^ and *Taxus wallichiana*^9^. In the former, the estimated divergence time between genealogies from the west and east side of Mekong-Salween Divide fell into the late Miocene, agreeing with the formation of the Mekong-Salween Divide. In another case, the present genealogical distribution of the plant species *Terminalia franchetii* was believed to be geographically structured by the paleo-drainage re-arrangements rather than the modern drainage systems, highlighting the role of historical geologic events in shaping contemporary genetic distribution^13^,^18^. A second known geological line is the “Tanaka-Kaiyong Line” (TKL), separating floristic subkingdoms into the Sino-Himalayan Forest (west) and Sino-Japanese Forest^19^,^20^. Molecular dating in several studies found the phylogeographic disjunction coincided well with the formation of this line (ca. 3 Mya), providing genetic evidence that geological processes might contribute to evolutionary history in plants^9^,^21^,^22^. Based on that evidence, the extensive uplifts of the QTP until ca. 4 Mya have been hypothesized to be the important force accelerating speciation^23^,^24^,^25^ and generating high biodiversity at both the inter- and intra-specific levels in the HHM region^9^,^10^,^26^,^27^,^28^.

In contrast to the geological hypothesis, the climatic hypothesis attributes population genetic diversification and species diversity mainly to Pleistocene glacial cycling. Specifically, it is hypothesized that Pleistocene glaciers may have isolated populations into glacial refugia and promoted population differentiation and intra-specific diversification without apparent geological breaks^1^. Fossil and moraine records show that the QTP and the adjacent HHM were subjected to a series of glaciations in the Pleistocene^29^,^30^. The ice sheet advanced and receded repeatedly during glacial-interglacial cycles, creating temporal physical barriers and dynamic heterogeneous niches. This process is believed to have facilitated genetic divergence^1^,^3^. Under this hypothesis, two different scenarios were proposed in recent molecular phylogeographic studies. The first scenario is high-altitude adaptation, in which some plant species survived the glacial stages at high altitude areas *in situ*^26^,^31^,^32^,^33^ and developed accompanying deep allopatric lineages with the Pleistocene glaciers^11^,^26^,^33^, even without large unified ice-sheets existing in the late Pleistocene^14^,^29^. The second scenario is migratory colonization, in which some plants in refugia migrated to low-altitude ice-free areas (e.g., the east edge of the QTP and the Hengduan Mountains) and recolonized the inner QTP platform during interglacial or postglacial stages^2^,^34^,^35^,^36^. The Hengduan Mountain region were proposed as an important refugium to the QTP and neighboring areas^10^,^36^, and several case studies have suggested that mountain glaciers in the Pleistocene rather than geological breaks resulted in allopatric divergence and profoundly affected the intraspecific phylogeographic structure^10^,^37^.

The two contrasting hypotheses to explain the causes of the high biodiversity in the HHM region have each been supported by a number of studies. However, they are not necessarily incompatible with each other. The effects of both historical events (geological and Pleistocene climatic events) may be tracked through the genomic imprinting of extant species in phylogeographic studies. Interestingly, up to now, most phylogeographic studies have focused on plants with shallow lineages, and the evolutionary history of these lineages primarily reflects the effects of Pleistocene climate changes^34^,^35^,^38^. Plants originating before the Pleistocene could provide good opportunities to infer the evolutionary forces due to both geologic changes in the Miocene and subsequent Pleistocene climate changes that occurred after their speciation. In this study, we studied the phylogeography of an alpine plant, *Oxyria sinensis* Hemsley (Polygonaceae), which diverged from its sole sister species *O. digyna* in the genus *Oxyria* between 12 and 14 Mya^27^. Because the TKL line runs primarily from ca. 33 ° N/102 ° E to 19 ° N/108 ° E in the Yunnan and Sichuan provinces^19^,^20^, it has seldom overlapped with the distribution of *O. sinensis*. Thus, in this study, we specifically aimed to test how the Mekong-Salween Divide formation and/or Pleistocene glacial-interglacial fluctuations affected the population history of *O. sinensis*.

In contrast to the worldwide distribution of *O. digyna*, *O. sinensis* is endemic to southwest China, with a center in the Hengduan Mountains, westward extension to the southeast Himalaya, and eastward extension to the east edge of the QTP (east QTP). There is a clear break in the distribution at the Mekong-Salween Divide, where the Mekong-Salween Divide appears to segregate *O. sinensis* into west and east populations (Fig. 1). Typical habitats of *O. sinensis* include mountain slopes, valleys and riversides, all at altitudes ranging from 1600 to 3800 m a.s.l. Considering the early origination and various ecological environments, *O. sinensis* must have experienced both of the above-mentioned historical events, making it an ideal species to test the evolutionary consequences of these events. In most angiosperms, nuclear DNA is biparentally transmitted and shows strong geographical homogenization because of extensive hybridization and/or introgression^3^. However, maternally inherited chloroplast DNA, which is dispersed through seeds without recombination, tends to be geographically structured. Chloroplast DNA has been demonstrated to be a powerful tool for tracing population history, founder effects, and demographic fluctuation^3^,^39^. In this study, we surveyed chloroplast (cp) DNA variations in *O. sinensis* using the *matK* fragment from the HHM region. We first estimated the genetic diversity and the divergence times of the different lineages detected. We next examined the population structure among different geographical groups. Then, we attempted to determine the possible factors driving the intraspecific divergence and the population structure. Finally, we used Bayesian inference methods and species distribution modeling to model the distribution changes of *O. sinensis* in response to Pleistocene climate changes. Thus, we had the following aims: 1) reveal the genetic diversity and geographic structure of contemporary *O. sinensis* populations; 2) reconstruct the demographic history of *O. sinensis*; 3) estimate the evolutionary consequences of the two historical events and assess their relative roles in shaping the contemporary genetic pattern of *O. sinensis*; and 4) infer the locations of the refugia in the Last Glacial Maximum (LGM) and regions of over-represented diversity that can help in the conservation and management of biodiversity in the HHM region.

## Results

### Phylogenetic history of *O. sinensis* and molecular dating

**Figure 1:**
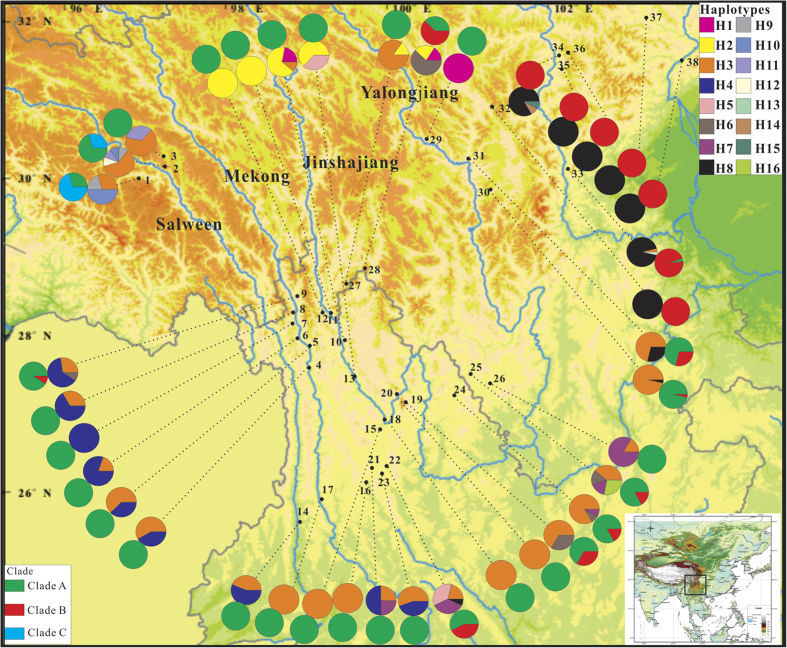
Sampling locations, geographical distribution of 16 matK haplotypes (H1 – H16) and three clades (**A**, **B**, **C**) identified by BEAST in *Oxyria sinensis* (population codes refer to Supplementary Table S1). The geographic groups for the Himalaya: population 1–3, and the Hengduan Mountains, population 4–28, and east QTP for population 29–38. Map was drawn using the R package “*Rgooglemaps*” and ArcGIS version 9.1 and modified using CorelDRAW X6.

We identified 16 distinct *mat*K haplotypes from 477 individuals in 38 populations (H1-H16; Supplementary Tables S1 & S2; Fig. 1). Among them, H3 was the most common, occurring in 22 of the 28 populations across the Himalaya, the Hengduan Mountains, and east QTP. Haplotype H8 was the second most common, occurring in ten populations, one in the Hengduan Mountains and nine in the east QTP. Six haplotypes (H5, H12–16) were unique to individual populations, and the remaining haplotypes occurred in more than one population. Fourteen populations (37.8%) had a single haplotype, and other populations (63.2%) harbored two or more haplotypes (Supplementary Table S1; Fig. 1).

**Figure 2:**
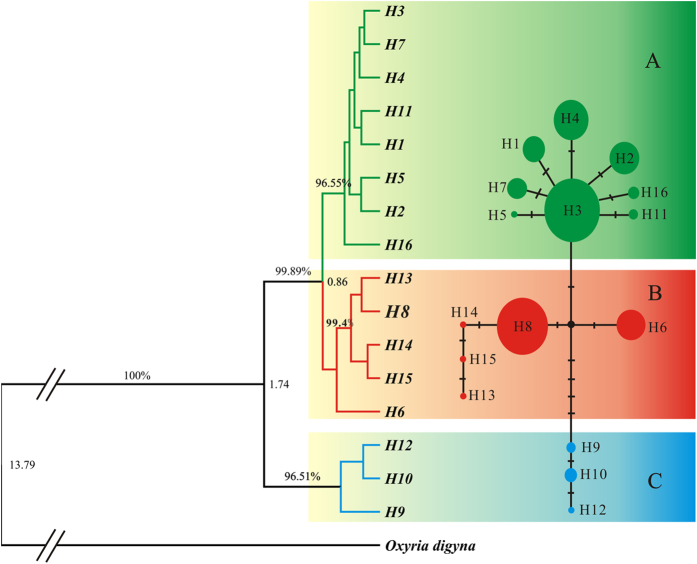
The Bayesian phylogenetic tree and minimum-spanning network constructed from all *mat*K haplotypes of *Oxyria sinensis*, with *O. digyna* as outgroup. Values above the tree branches represent the posterior probabilities from the BEAST Bayesian analysis and those at the tree nodes denote the divergence time in million-year units. The 95% HPD for is 13.79 Mya with 95% HPD from 7.54 to 24.63 Mya. The clades A and B diverged from clade C 1.74 Mya with 95% HPD from 0.72 to 3.46 Mya, and the divergence time between clades A and B occurred 0.86 Mya with 95% HPD from 0.34 to 1.73 Mya. In the haplotype network, filled circles represent unique haplotypes and their sizes correspond to their frequencies across all populations. Each crossed bar between two circles depicts one nucleotide difference between the two haplotypes. The map was generated from the BEAST program and modified using the CorelDRAW X6 software.

The distinct *mat*K haplotypes differed at 18 variable sites within the 1173-bp sequence alignment. All variations were polymorphic nucleotide substitutions, and no insertions or deletions were found. The Bayesian phylogenetic analyses identified three well-supported clades (named A, B, and C). Clade A appeared to be a widely distributed clade, consisting of 8 unique haplotypes that were widespread throughout the region. Broadly, Clade B contained 5 haplotypes, including an unresolved haplotype H6 (support value < 50%). After excluding the H6, Clade B was exclusively distributed in the east QTP. The H6 mainly occurred in the Hengduan Mountain region. Clade C contained three haplotypes, all of which occurred only in the Himalaya. The minimum-spanning network of these haplotypes produced a similar grouping pattern (Fig. 2).

The estimated divergence time between *O. sinensis* and *O. digyna* was approximately 13.79 Mya (95% HPD: 7.54–24.63 Mya) in the Miocene. This estimate is well consistent with an estimate based on the fossil record (12–14 Mya)^27^,^40^, suggesting that the substitution rates and models applied in this study were appropriate. Clades A and B diverged from C approximately 1.74 Mya (95% HPD: 0.72–3.46 Mya) in the early Pleistocene, and the recent divergence between clades A and B occurred approximately 0.86 Mya (95% HPD: 0.34–1.73 Mya).

### Genetic diversity

Genetic differentiation for the overall populations (*G*_ST_ = 0.611; *N*_ST_ = 0.662) was high. The high population differentiation was also supported by AMOVA and BARRIER analyses, as reflected by the distinct haplotype composition among the three geographic groups or the predicted phylogeographic groups. However, *N*_ST_ was not significantly higher than *G*_ST_ (*P *> 0.05), indicating a nonsignificant phylogeographic structure. This might be due to the discontinuous distribution of the shared dominant haplotypes (e.g., H3 and H8) across different geographic regions. Furthermore, BARRIER predicted that a major phylogeographic structure existed between the HHM and the east QTP (Supplementary Fig. S2). AMOVA analysis revealed that genetic differentiation across all populations accounted for 66.82% of the total variation, whereas among three geographic groups (Himalaya, Hengduan Mountains and east QTP), the variance components accounted for a slightly higher proportion of variation (38.95%) compared with the within-population among groups and the within-population variation (Table 1). Theoverall genetic diversity was very high (*H*_T_ = 0.791), contrasting the low degree of within-population diversity (*H*_S_ = 0.308). The haplotype diversity (*h*_*e*_) within populations varied from 0.000 to 0.833 (Table 1; Supplementary Fig. S1). We also computed the average haplotype diversity for each group. The highest diversity (0.6297) was found in the Himalaya, with an intermediate value in the Hengduan Mountains (0.3522), and the lowest value in east QTP (0.1005).

### Population demographic expansion

**Figure 3:**
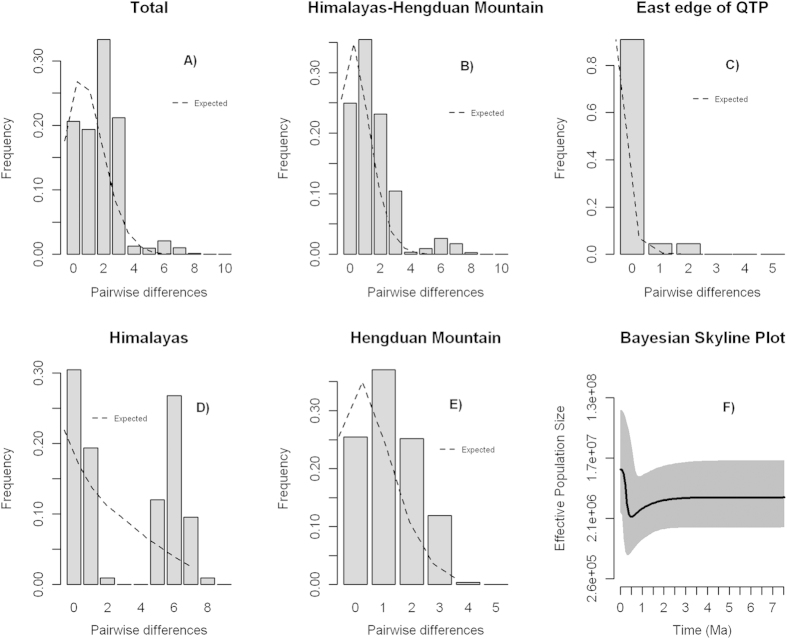
Mismatch distributions (**A–D**) and the Bayesian skyline plot (**E**) of *mat*K sequence data from four different regions. Except for the populations in east Himalaya, all other regional populations showed unimodal mismatch distributions, a sign of population expansion. In the Bayesian skyline plot, the estimated effective population size (*N*e) is depicted on the y-axis is on a *log*2 scale. The median estimate of *N*e is shown by the thick black line, and the 95% highest probability density (HPD) of posterior distribution of *N*e is marked in gray.

We investigated the population demographic history of *O. sinensis* for the three focal geographic regions: Himalaya, Hengduan Mountains and the east QTP. The Himalaya populations (1–3) showed a multimodal mismatch distribution (Fig. 3), suggesting that no historical expansions occurred in this region. By contrast, unimodal mismatch distributions were found from the Hengduan Mountains and east QTP (Fig. 3), which did not differ significantly from the expected distribution under a sudden population expansion model (P > 0.05, Table 2). In addition, theneutrality test for the Hengduan Mountains and east QTP groups showed negative Tajima’s *D* and Fu’s *Fs* values, though these were not statistically significant (except Fu’s *Fs* for east QTP, P < 0.001; Table 2), suggesting that the populations in these regions deviated from the neutral equilibrium population model. The expansion time estimated for the Hengduan Mountains and the east QTP based on mismatch analysis was approximately 0.14 and 0.31 Mya, respectively (Table 2). A Bayesian skyline plots (BSPs) analysis of total populations indicated a slight decline in population size since 0.8 Mya and an approximately five-fold rapid increase since 0.3 Mya (Fig. 3), which is consistent with the mismatch analysis.

### Species distribution modeling in the present and the past

We reconstructed the glaciers under present conditions and the LGM. Because there is still no consensus about the exact locations and range of glaciers at the LGM in the QTP, we used the annual mean temperature layer from both ecological conditions. It is believed that the ice sheet might have reached 2000 meters in thickness and covered the entire plateau regions at an altitude above 3000 meters^41^. In general, our reconstructed glaciers are congruent with those of Ehlers and Gibbard^42^, who use minimum-glaciations range reconstruction for glacial geology. Most glaciers run along the mountains ridges in the HHM, indicating that mountain glaciers may have played an important role in contributing to the population structure of *O. sinensis*.

**Figure 4:**
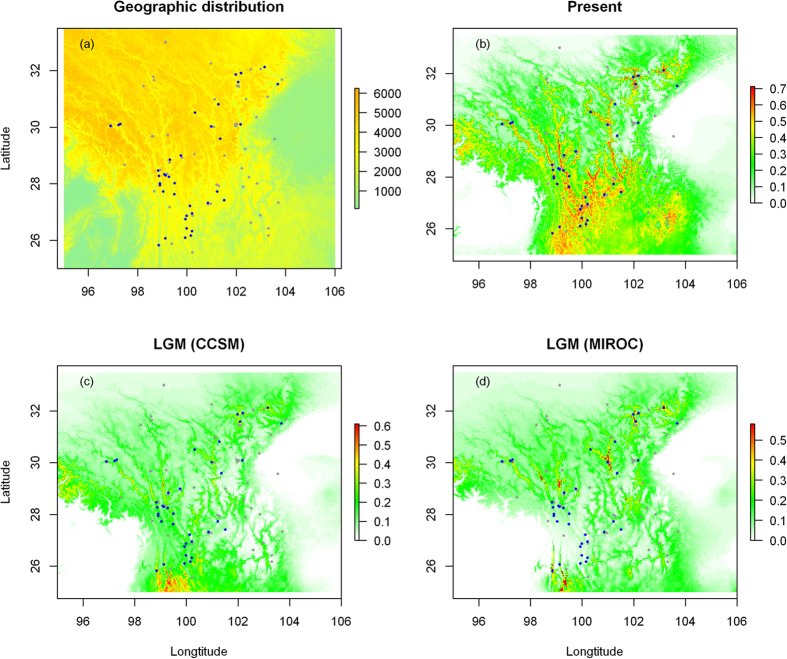
Distribution records locations, and species potential distribution of *O. sinensis* under current and projected to past climate conditions. (**a**) The distribution records used for SDM were plotted from both this study and the CVH. We obtained the 30 arc-second resolution for altitude data from WorldClim (http://www.worldclim.org/current). This map provides the information about the locations of mountains and the occurrences of *O. sinensis*. The predicted species occurrences were obtained with an ensemble-forecasting approach: (**b**) current climate, (**c**) the LGM climate for CCSM, and (**d**) the LGM climate for MIROC. The color gradient from white to green and red indicates the suitability levels from low to high. The blue points depict the sampling location in this study, and gray points represent specimen records from the CVH.

**Figure 5:**
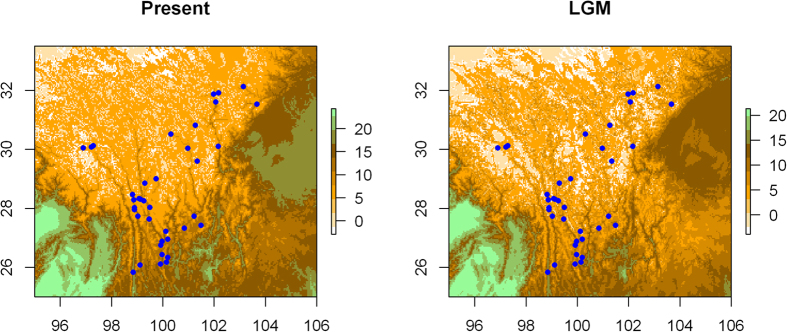
Glacier locations for current and the LGM. (**a**) The glaciers in present conditions and (**b**) the glaciers in LGM. Because there is no consensus about the exact locations and range of glaciers at LGM on the QTP, we reconstructed the glaciers for both LGM and the present using the Annual Mean Temperature. The sampled *O. sinensis* between latitude 29° and 32° were surrounded by mountain glaciers, indicating that mountain glaciers played an important role in shaping the population structure.

Species distribution models (SDM) were reconstructed for the present and the LGM (Fig. 4b–d). Model performance showed that all models met the threshold for inclusion in the ensemble, with all three statistical scores > 0.72, indicating good model performance. The realized present distribution of *O. sinensis* was generally well recovered by the consensus models (Fig. 4b). Projection to the environmental niche of the LGM indicates suitable habitats essentially located within the mountain valley of the HHM, a low altitude area of south Hengduan Mountain and east QTP (Fig. 4c,d). A high concordance between the suitable climate areas under the modeled LGM and the present was found (Schoener’s *I* = 0.9585, *D* = 0.7936 for CCSM; Table 3), suggesting a stable distribution of *O. sinensis* over the last 21,000 years. The SDM uncovered a dynamic history of geographic shifts in suitable areas for *O. sinensis* (Fig. 4b–d), with mountain valleys and low-altitude regions maintaining suitable habitats, possibly serving as refugia in the glacial periods throughout the glacial-interglacial cycles (Fig. 5).

## Discussion

Recently, several studies in plants found that phylogeographic disjunction coincided with the formation of the Mekong-Salween Divide^9^,^10^,^16^. Our data do not conform to this scenario but indicate that the main chloroplast genetic divergence of *O. sinensis* coincided with several Pleistocene glacial stages. Much of the variation within the Himalaya and east QTP is likely due to isolation and population expansions induced by glacial–interglacial cycles. In the following sections, we first discuss Pleistocene climate changes rather than the formation of Mekong-Salween Divide as the possible primary evolutionary factors driving intraspecific divergence. We then highlight the impact of Pleistocene glacial-interglacial cycles and range expansions on the maternal evolutionary history of *O. sinensis* and further discuss the implications of our findings for conservation biology.

### Refugial isolation vs. geological isolation

In this study, we explored the phylogeography of *O. sinensis.* The estimated divergence time between *O. sinensis* and its closely related species *O. digyna* was approximately 13.8 Mya (Fig. 2). Although the time calibration must be interpreted cautiously, our estimated speciation timing of *O. sinensis* was well consistent with dating based on fossils from a recent study^27^. Since *O. sinensis* diverged from its sibling species *O. digyna* approximately 13.8 Mya (Fig. 2), it must have experienced both Pleistocene climate changes and the formation of the Mekong-Salween Divide. Both historical events presumably contributed to population isolation and thus to allopatric genetic differentiation between isolated populations. However, our phylogenetic analyses identified three major clades (Fig. 2), and those clades show spatial clustering into three primary geographic regions. In contrast to early origination, relatively shallow intraspecific lineages were found in our study (Fig. 2). The divergence time for the three major clades was approximately 1.74 to 0.86 Mya, which falls into the early or middle Pleistocene (Fig. 2). The age of the deepest lineages of *O. sinensis* clearly post-dates the formation of a known geological break, the Mekong-Salween Divide, which arose in the late Miocene and early Pliocene^9^,^10^. In addition, BARRIER analysis found that a major phylogeographic disjunction occurred between the HHM and east QTP, not following the Mekong-Salween Divide. Taken together, these findings suggest that geological isolation might not be the main physical factor driving lineage divergence in *O. sinensis*.

By contrast, the estimated divergence times for intraspecific lineages are in concordant with two glacial events: the Eburonian glacial stage (1.2–1.7 Mya) and the Maximum glacial stage (0.6–0.8 Mya) in the early and/or middle Pleistocene^43^. On the QTP, the Pleistocene glaciers reached a maximum altitude during the maximum glacial stage^30^,^44^,^45^. Ice covered five to seven times more area than at present, and extensive mountain glaciers developed in the eastern Himalaya and Hengduan Mountains^30^,^45^,^46^,^47^. Therefore, we suppose that these mountains may harbor individuals of *O. sinensis* that survived *in situ* for a long time and that the temporary mountain glaciers might have acted as physical barriers of gene flow and promoted fragmentation and genetic differentiation. Given a sufficiently long time, population fragmentation and genetic differentiation eventually arose. Although a number of sequential glaciers occurred after the maximum glacial stage, they had minor impacts on the extant spatial genetic distribution of *O. sinensis*^14^. Bearing this in mind, we propose that the development of mountain glaciers during early/middle Pleistocene glacial stages mostly likely served as the main factor causing lineage divergence of *O. sinensis* due to its relatively low intensity. In agreement with this study, Fan *et. al*.^37^ also found that climate changes (monsoon) associated with the formation of TKL rather than TKL rising drove the spatial genetic structure of *Sophora davidii*. Deep lineages and similar diversification patterns due to Pleistocene climate changes were also found in other plants in the QTP region^10^,^26^,^31^,^33^.

### Multiple glacial refugia and demographic history

Glacial refugia were recognized as harboring high intraspecific diversity and major lineages^1^. The identification of three phylogenetic lineages suggests that at least three historical refugia existed in the followed recurrent cycles of Pleistocene cooling and warming since the origination of *O. sinensis*. The Himalaya harbored three haplotypes on the basal clade, representing the eldest refugium. A number of populations which harbor high genetic diversity and abundant unique haplotypes were potential refugia during the cooling stages (Fig. 1; Supplementary Fig. S1); for example, the Himalaya populations, the central Hengduan Mountains (e.g., populations 10, 13 and 28), the southern Hengduan Mountains (e.g., populations 21–23), and the eastern QTP (e.g., population 31, 34). Given the early and middle Pleistocene origination of these unique haplotypes (Fig. 2), it is reasonable that these populations have served as glacial refugia in the cooling stages since their origination and expanded their range rapidly during the following interglacial stages. The SDM also predicted suitable habitats of *O. sinensis* sundered by high mountains resulting in multiple centers of genetic diversity, reflecting the geographic isolates (Fig. 4b–d). Therefore, *O. sinensis* likely survived in those multiple locations *in situ* during the LGM. The SDM predicted that the most suitable habitats under present conditions were highly concordant but slightly larger than during the LGM (Fig. 4b,c) and that the present mountain glaciers were highly similar to those in the LGM but slightly smaller at present (Fig. 5). These results suggest that *O. sinensis* seldom changed its distribution since the LGM. This also indicates that mountain glaciers in this region have not greatly expanded but have slightly shrunk since the LGM. Overall, our findings suggest that the mountain glaciers and range expansions in early and middle Pleistocene had a predominant effect on the spatial genetic structure of *O. sinensis*. The fact that suitable areas have changed relatively little between the LGM and the present indicates that *O. sinensis* has only been slightly affected by ice-sheets and/or mountain glaciers since the LGM^48^,^49^,^50^. This finding supports the theory that no large ice sheet/mountain glacier developed in the Hengduan Mountains region during this period.

The duration of mountain glaciers plays an important role in lineage and population divergence. However, rare gene exchanges most likely occurred among neighboring regions during the retreat of the glaciers. The Himalaya group harbored three haplotypes, all of which located on basal clade C and may represent the earliest diversification center. The presence of haplotype H3 in the Himalaya region could be due to occasional gene flow from the Hengduan Mountains and might have been achieved through dispersion along the Salween River valley. Such long-distance dispersion might also occur between the Yunnan and Sichuan ranges, leading to unclear boundaries between the Hengduan Mountains and east QTP (Fig. 1). Those long-distance migration patterns were also found in *Sinopodophyllum hexandrum*^10^, an alpine herb distributed sympatrically with *O. sinensis*. An alternative scenario is that those shared haplotypes might be relics and/or close derivatives of ancient haplotypes due to an ancient common ancestry and incomplete lineage sorting. In the network topology, we observed a couple of intermediate states, which may represent those missing ancestral haplotypes and/or unsampled haplotypes.

The estimates of *G*st and *N*st (*G*_ST_ = 0.6110; *N*_ST_ = 0.6620) indicate high population differentiation across the entire distribution of *O. sinensis*, but no significant phylogeographic structure was found based on a comparison of *G*_ST_ and *N*_ST_ (P > 0.05). The high geographic genetic differentiation primarily reflects the composition of the distinct haplotypes among the three geographic groups or the phylogeographic disjunction between the HHM and east QTP identified from BARRIER. The nonsignificant phylogeographic signal most likely occurred because of the discontinuous distribution of the common haplotypes (e.g., H3 and H8) across different geographic regions. We detected the signatures of repeated regional expansions based on a combination of multiple statistical analyses, including Fu’s *F*s and Tajima’s *D* statistics, BSPs, and hierarchical mismatch distribution. BSPs indicated that the population size had increased dramatically since 0.5 Mya (Fig. 3f). Further hierarchical mismatch analyses suggested at least two independent population expansions occurred, and the expansion times were dated to before LGM. The first expansion led by haplotype H8 occurred mainly in the east QTP approximately 0.3 Mya and fell into the interglacial period after the Maximum glaciations on the QTP^29^,^46^,^51^. The second expansion occurred approximately 0.14 Mya during the Last Interglacial period (LIG, 0.12–0.14 Mya)^14^,^29^,^44^,^45^, during which the populations recolonized either westward to Himalaya or eastward to the east QTP. These multiple expansions may have reshuffled the genetic distribution in geography, which, together with genetic drift, led to homogeneity within geographic groups. For example, in east QTP, more than half of the populations were fixed with H8, and more than ten populations (>50%) had H3. The repeated range expansions led to the homogeneity within geographic groups, and subsequent secondary contacts may further blur the boundaries among geographic groups, resulting in inconsistency of two major phylogeographic groups (predicted by BARRIER) and three geographic groups. Taken together, these multiple lines of evidence provide a good understanding of the population history of *O. sinensis*; however, more comprehensive knowledge should be gained through the use of multiple markers, such as nuclear DNA markers. Similar pre-LGM demographic expansions were reported for other plants in the QTP, such as *Pedicularis longiflora*^36^, *Potentilla glabra*^31^ and *Hippophae tibetana*^52^.

### Implications for management of biodiversity conservation

The understanding of the geographical distribution of cpDNA variations may be used for practical applications, such as preventing the loss of genetic resources, conservation management and breeding strategy. More than 12,000 plant species inhabit in the HHM, and a large proportion is endemic. Efforts to identify localities harboring distinct genetic genealogies and regions rich in biodiversity will provide valuable resources for conservation management in this worldwide biodiversity “hotspot” region. Our genetic survey has at least two important implications. First, the three geographic regions (the Himalaya, Hengduan Mountains and east QTP) could be recognized as distinct genetic units, and merit further conservation recognition from a management perspective. Secondly, we found that the Himalaya harbor the highest genetic diversity, whereas the Hengduan Mountains harbor moderate diversity. This poses an urgent need that is of the highest priority for the conservation management. These locations include the Himalaya (populations 1–3), the central Hengduan Mountains (e.g., 10, 13 and 28), the south Hengduan Mountains (e.g., 21–23), and the east QTP (e.g., population 31, 34). In addition, we should note that only a few field samples were obtained in the Himalaya, implying a rather small natural population and a potential loss of genetic diversity of *O. sinensis*. The future conservation policy for *O. sinensis* should focus on protection of regions of distinct lineages and high diversity. The most effective conservation strategy may combine sampling populations containing different genealogies and field protection of those rich biodiversity regions *in situ*. Overall, our findings revealed the genetic structure of *O. sinensis* and tentatively indicated geographic regions with excess genetic diversity. These findings could facilitate short-term and long-term conservation in this worldwide biodiversity “hotspot” region.

## Conclusions

For plants originated in the Miocene on the QTP, their genetic structure could have been affected by the geological changes due to uplifts of QTP and glacial-interglacial oscillations during the Pleistocene since their speciation. Our phylogeographic analysis of *O. sinensis* suggests that Pleistocene refugial isolation may have driven lineage divergence at spatio-temporal timescales and that later interglacial range expansions blurred the phylogeographic boundaries created in the early Pleistocene. Our results provide one of the few detailed maternal evolutionary histories of endemic plants with deep lineages and give important insight into the origin and maintenance of genetic diversity in a global biodiversity “hotspot” region.

## Materials and Methods

### Plant material

*Oxyria sinensis* is a dioecious perennial herb endemic to the Himalaya-Hengdua Mountains (HHM) that can reproduce sexually via seeds and asexually via rhizomes^53^. This plant is characterized by tiny flowers, densely branched panicles, and winged achenes that favor potential dispersal by wind. Samples of *O. sinensis* were collected from 38 populations across its entire range between 2008 and 2011 (Fig. 1 and Supplementary Table S1), representing three broad geographical groups: the Himalaya, the Hengduan Mountains and the east Qinghai-Tibetan Plateau (QTP). A total of 477 individuals were sampled, with an average of 12 individuals per location (Supplementary Table S1). By comparing records from the Chinese Virtual Herbarium (CVH; http://www.cvh.ac.cn/), our sampling generally reflects the overall distribution of *O. sinensis* (Fig. 4a; Supplementary Table S3). All samples were stored at -20 °C in the herbarium of the School of Life Science, Yunnan Normal University, P. R. China.

### DNA extraction, amplification and sequencing

Genomic DNA was extracted from 20 mg of dried leaf tissue from each *O. sinensis* individual, using a QIAGEN DNeasy Tissue Kit (Qiagen, BOSITE Biology Co. Ltd., Shanghai, China) following the manufacturer’s protocol. We amplified the chloroplast Muturase K (*mat*K) gene using polymerase chain reactions (PCR), with a primer pair of matKAF (Forward, 5’-CTA TAT CCA CTT ATC TTT CAG GAG-3’) and matK8R (Reverse, 5’-AAA GTT CTA GCA CAA GAA AGT CGA-3’). The PCR reactions were performed in a 25 μL reaction volume, containing 1.5 μL genomic DNA, 50 mM Tris-HCL, 1.5 mM MgCl_2_, 250 μg/mL bovine serum albumin (BSA), 0.5 mM dNTPs, 0.2 μM of each primer, and 0.75 units of *Taq* polymerase. Programmed amplification parameters were as follows: 4 min denaturation at 94 °C, then 35 cycles of 50 sec denaturation at 94 °C, 50 sec annealing at 53 °C, and 1 min 30 sec extension at 72 °C, and a final extension of 7 min at 72 °C. PCR products were purified before sequencing to remove excess primers and deoxynucleotide triphosphates using a TIAN quick Midi Purification Kit (Tiangen Biotechnology Co. Ltd., Beijing, China). Sequencing reactions were performed using ABI Prism Sequencing Ready Reaction Kit with the same primers as PCRs, and analyzed on the ABI 3730 genetic analyzer (Applied Biosystems).

### Phylogenetic analysis and molecular dating

DNA sequences were aligned using CLUSTAL X^54^ with default parameters and checked manually. To root a phylogenetic tree, we additionally sequenced the *mat*K gene from a closely related *Oxyria* species (*O. digyna*)^40^,^55^ and used this sequence as the outgroup for all the *O. sinensis* sequences. All sequences generated in this study have been deposited in GenBank under accession numbers KJ159010–KJ159025.

The genealogical relationships among *mat*K haplotypes and divergence times were jointly estimated through a Bayesian Markov Chain Monte Carlo approach implemented in BEAST v.1.7.2^56^. Because the substitution rate of chloroplast DNA (cpDNA) for *Oxyria* is unknown, we used a range of mutation rates from 1.01 × 10^−9^ to 2.9 × 10^−9^ substitution/site/year (s/s/y) that are believed valid for chloroplast DNA of angiosperms overall^57^,^58^,^59^. The divergence time of each bifurcating event was estimated under the strict molecular clock model, assuming no rate variation across the tree branches. We used jModeltest v2.1.4^60^,^61^ to determine the best-fitting DNA substitution model of sequence evolution using the Akaike Information Criterion (AIC) and comparing –*ln* likelihood scores. The GTR + I model was chosen and applied to all Bayesian inferences. Three independent Markov Chain Monte Carlo (MCMC) runs were performed, with each run starting a random tree for 200,000,000 generations, and sampled for every 1000 generations. Convergence of three independent runs was evaluated using Tracer v.1.5 (http://evolve.zoo.ox.ac.uk/), with an effective sample size (ESS) for all parameters larger than 100. The results in log files of multiple runs were combined using LogCombiner v.1.5.4^62^ and analyzed collectively. The first 20% of the generations were discarded as burn-in, and the rest were retained as valid samples for further analysis. A maximum clade credibility tree and node-specific parameters were computed from the sampled data using TreeAnnotator (http://beast.bio.ed.ac.uk/TreeAnnotator/). The tree was plotted using FigTree v.1.31 (http://tree.bio.ed.ac.uk/software/figtree/) and further graphically modified for publication using graphic design software CorelDRAW X6 (Corel Corp., Ottawa, Canada).

To explore the genealogical relationships and their phylogeographic pattern, we also constructed the parsimonious networks for all *mat*K haplotypes of *O. sinensis* using TCS 1.21^63^, under a 95% criterion of statistical parsimony^64^.

### Phylogeographic and population genetic analyses

The average haplotype diversity within populations (*H*_S_) and among populations (*H*_T_), and two population differentiation parameters, *G*_ST_ and *N*_ST_, were computed using the program PERMUT with 1000 permutations^65^ (http://www.pierroton.inra.fr/genetics/labo/Software/PermutCpSSR). Geographic structure and genetic differentiation within and between geographical regions were statistically tested through analyses of molecular variance (AMOVA)^66^ in ARELQUIN v3.1.1^67^. The haplotype diversity (*H*_*E*_) and nucleotide diversity (π) for geographic populations were also calculated using ARLEQUIN.

Demographic dynamics through time were inferred using Bayesian skyline plots (BSPs) in BEAST. The BSPs employ a coalescent approach to estimate the effective population size and its changes over piecewise coalescent intervals^68^. The BSPs estimates were obtained through MCMC runs in BEAST, using the same running parameters for phylogenetic and clock time inference described above, including a DNA substitution model, starting tree, running length, and burn-in length.

Historical population expansions were tested using mismatch distribution analysis implemented in ARLEQUIN. The mismatch analysis compares the observed frequency distribution of pairwise differences among haplotypes with the expected from those under the parametric demographic expansion model. A uni-modal distribution of pairwise haplotypes difference is expected if a population has undergone a recent expansion, whereas a multimodal distribution suggests a population in equilibrium^69^. Evidence of recent demographic expansion was also examined using Harpending’s Raggedness index^70^, Tajima’s *D*^71^ and Fu’s *F*_S_ test^72^. For a population fitting a sudden expansion model, expansion time (*t*, in years) was estimated from the mutation rate (*u*) and number of generations since expansion (τ), using Rogers and Harpending’s^70^ approach, τ = 2*ut*. The mutation rate *u* was calculated from the DNA substitution rate (*μ*), sequence length (*k*) and the generation time (*g*) of *O. sinensis* using the formula *u* *=* 2*μkg*. A generation time of 2 years and an average *cp*DNA mutation rate of 2 × 10^−9^ s/s/y were assumed^57^,^58^,^59^.

BARRIER v2.2^73^ was used to predict the potential phylogeographic boundaries exhibiting the largest genetic discontinuities among all population pairs. The program uses a genetic matrix of pairwise Fst values from all of the geographic population pairs to construct a geometric network of populations. Putative genetic boundaries were further identified based on Monmonier’s maximum difference algorithm^73^.

### Species distribution modeling

Species niche modeligng (SDM) was conducted to examine the influence of Pleistocene climate changes on the geographic distribution of *O. sinensis*. Environmental conditions including 19 bioclimate variables were downloaded from the WorldClim database (http://www.worldclim.org). Bioclimate layers were obtained in the 2.5 arc-minute resolution for the present and the LGM (ca. 21,000 years ago). Climate data from two general circulation model (GCM) simulations from the Community Climate System Model (CCSM)^74^,^75^ and the Palaeoclimate Modeling Intercomparison Project (MIROC)^76^, were used in this study.

Distribution records of *O. sinensis* were compiled from our field study and the Chinese Virtual Herbarium (CVH; http://www.cvh.ac.cn/). We obtained 205 hits by searching “*Oxyria Sinensis*” in the CVH (last visit on 10/10/2014), representing historical collections from 1906 to present. Exact geographic information was obtained from 81 records, i.e., the longitude and latitude coordinates were recorded by collectors. For those without geographical coordinates, we mapped their locations to the nearest county. After removing duplicates, we obtained 64 additional unique records from CVH. After merging the CVH and this study, a total of 102 records were included and subjected to the SDM.

We applied three algorithms to build the SDM – one regression methods: generalized linear models (GLM) and two machine learning methods: random forests (RF) and maximum entropy (MAXENT). Models were created for each algorithm to predict the effects of topography on species distribution. All three algorithms were implemented in “*Biomod2*” (http://cran.r-project.org/web/packages/biomod2/index.html) packages in R v. 3.0 (http://www.R-project.org/). For the MAXENT method, Maxent v 3.3.3k^77^ was downloaded from http://www.cs.princeton.edu/~schapire/maxent/. Because all three methods require presence-absence data and *O. sinensis* lacks true absences records, we randomly generated 10,000 background pseudo-absence points. All three SDM modeling approaches were combined to construct the realized distribution model using an ensemble forecasting (EM) techniques to account for prediction uncertainty among different algorithms and outperform single models^78^,^79^.

To evaluate model predictive performance, we randomly split the original dataset into two subsets, using 70% (training dataset) to calibrate each model and 30% (testing dataset) for model evaluation. We repeated the processes 30 times to obtain robust estimates of the SDM. The accuracy of the model performance was evaluated using three indices: the receiver operational characteristic (ROC), the True Skill Statistic (TSS) and Cohen’s Kappa (KAPPA). The ROC curve plots with true positive rate of model simulation as a function of the false positive rate^71^. The measure of the area under the ROC curve (AUC) measures the area under the curve, providing a threshold independent accuracy index. The AUC values vary from 0 to 1, with a higher value indicating a more fitting performance. An AUC of greater than 0.5 suggests that the given model has non-random discrimination abilities. The TSS statistic measures the agreement between the expected and observed distribution and is not influenced by prevalence. TSS ranges from –1 (perfect disagreement) to 1 (perfect agreement), and a TSS value less than 0 generally indicates the model’s predictive performance is no better than random. The KAPPA statistic is closely related to TSS, taking into account agreement between the observed and the expected distribution by chance.

We obtained the projections for the LGM by applying the realized models fitted to the present conditions of *O. sinensis*. To compare the similarity of niches (or niche overlap) between the two ecological layers, we computed Schoener’s *D*^80^ and *I* statistics^81^ using the ENMTOOLS^82^,^83^. The values of the two parameters vary between 0 (no overlaps at all) and 1 (complete overlapping), with a fraction number indicating a certain level of similarity in ecological niches. Finally, we visualized the predicted species distribution using DIVA-GIS v.7.5 (http://www.diva-gis.org/) and R 3.0.

## Additional Information

**How to cite this article**: Meng, L. *et al.* Refugial isolation and range expansions drive the genetic structure of *Oxyria sinensis* (Polygonaceae) in the Himalaya-Hengduan Mountains. *Sci. Rep.* **5**, 10396; doi: 10.1038/srep10396 (2015).

## Supplementary Material

Supplementary Information

## Figures and Tables

**Table 1:** Results of the analyses of molecular variance (AMOVA) for *O. sinensis*. Overall, genetic differentiation was examined for a total of 477 individuals from 38 geographic populations, as well as hierarchical geographic structures tests for three geographic groups: the Himalaya, the Hengduan Mountains and east QTP.

**Source of variation**	**df**	**Sum of Squares**	**Variance Components**	**Variation (%)**	**Fixation index**
***All***					
Among Populations	37	294.526	0.6196 Va	66.82	*F*st = 0.6682***
Within Populations	439	135.047	0.3076 Vb	33.18	
***Himalaya (1–3) vs. Hengduan Mountains (4–28) vs. east QTP (29–38)***					
Among groups	2	123.615	0.4355 Va	38.95	*F*sc = 0.5512***
Among populations within groups	35	170.911	0.3778 Vb	33.71	Fst = 0.7256***
Within populations	439	135.047	0.3076 Vc	27.44	*F*ct = 0.3885***

All tests were based on both molecular distances and haplotype frequencies. Statistical significance: P < 0.001 (***) and P < 0.01 (**).

**Table 2:** Neutrality and mismatch analyses for the *O. sinensis* populations and four designated regional groups,

	***N***	**τ**	**Θ**_**0**_	**Θ**_**1**_	***SSD***	***RAG (p)***	***Tajima’s*** **D**	***Fu’s*****Fs**	***Expansion Time (year)***
Himalaya	26	7.2813	0.0000	3.6719	0.0919	0.1199 (0.2980)	1.5950	2.7058	−
Hengduan Mountains	321	1.3457	2.0000	99999	0.0018	0.0585 (0.1480)	−0.4614	−0.7633	143404
East QTP	130	3.0000	0.0000	0.0808	0.0024	0.7502 (0.7670)	−1.5444*	−5.2660**	319693
Total	477	2.0703	0.0211	11.7047	0.0207*	0.0744 (0.1230)	−0.7842	−2.9243	220621

Note: *N*, sample size; *τ*, time in generation unit since the population expansion happened; *θ*_0_ and *θ*_*1*_, nucleotide diversity measures prior to and posterior to expansion scaled with population size; RAG, the Harpending’s Raggedness index; *a*, H3 was excluded for the calculation; SSD, sum of squared deviations.

**Table 3:** Degree of niche overlap based on Schoener’s *I* and *D* statistics. The lower triangles represent the *D* value and the upper represent the *I* value. The statistics indicate high identity among suitable habitats among all pairwise comparisons.

***D\I***	**Present**	**LGM (CCSM)**	**LGM (MIROC)**
Present	−	0.9585	0.9405
LGM (CCSM)	0.7936	−	0.9627
LGM (MIROC)	0.7447	0.8063	−
