# Supplementary material for: Refugial isolation and range expansions drive the genetic structure of Oxyria sinensis (Polygonaceae) in the Himalaya-Hengduan Mountains
Source: Sci Rep. 2015 May 27;5:10396. doi: 10.1038/srep10396 (PMC4445061; doi:10.1038/srep10396)
Supplement: Supplementary Information [file srep10396-s1.doc]

Title: Refugial isolation and range expansions drive the genetic structure of Oxyria sinensis (Polygonaceae) in the Himalaya-Hengduan Mountains

Author: Lihua Meng, Gang Chen, Zhonghu Li, Yongping Yang, Zhengkun Wang, Liuyang Wang

**Table S1**. Geographic information of sampled *O. sinensis* populations, haplotype distribution, gene diversity (*He*) and nucleotide diversity (πe) for each population.

| P. | Location | Latitude | Longitude | Alt.(m) | N | H1 | H2 | H3 | H4 | H5 | H6 | H7 | H8 | H9 | H10 | H11 | H12 | H13 | H14 | H15 | H16 | *H_e_* | *π_e_* |
| --- | --- | --- | --- | --- | --- | --- | --- | --- | --- | --- | --- | --- | --- | --- | --- | --- | --- | --- | --- | --- | --- | --- | --- |
| 1 | Basu, TB | 30°03′N | 96°54′W | 3234 | 10 |  |  | 3 |  |  |  |  |  | 2 | 5 |  |  |  |  |  |  | 0.689 | 0.003 |
| 2 | Tongni, TB | 30°05′N | 97°13′W | 2739 | 10 |  |  | 6 |  |  |  |  |  | 1 | 1 | 1 | 1 |  |  |  |  | 0.667 | 0.003 |
| 3 | Bangda, TB | 30°07′N | 97°18′W | 3600 | 6 |  |  | 4 |  |  |  |  |  |  |  | 2 |  |  |  |  |  | 0.533 | 0.001 |
| 4 | Yezhi, YN | 27°44′N | 99°02′W | 1789 | 12 |  |  | 7 | 5 |  |  |  |  |  |  |  |  |  |  |  |  | 0.530 | 0.001 |
| 5 | Badi, YN | 27°57′N | 98°54′W | 1837 | 8 |  |  | 5 | 3 |  |  |  |  |  |  |  |  |  |  |  |  | 0.536 | 0.001 |
| 6 | Cizhong, YN | 28°01′N | 98°54′W | 1953 | 15 |  |  | 3 | 12 |  |  |  |  |  |  |  |  |  |  |  |  | 0.343 | 0.000 |
| 7 | Yunling, YN | 28°17′N | 98°52′W | 2150 | 9 |  |  |  | 9 |  |  |  |  |  |  |  |  |  |  |  |  | 0.000 | 0.000 |
| 8 | Deqin, YN | 28°20′N | 99°05′W | 4307 | 9 |  |  | 3 | 6 |  |  |  |  |  |  |  |  |  |  |  |  | 0.500 | 0.000 |
| 9 | Zhongdian, YN | 28°28′N | 98°50′W | 2965 | 11 |  |  | 3 | 7 |  | 1 |  |  |  |  |  |  |  |  |  |  | 0.564 | 0.001 |
| 10 | Nixi, YN | 28°02′N | 99°30′W | 3139 | 18 | 4 | 12 | 2 |  |  |  |  |  |  |  |  |  |  |  |  |  | 0.523 | 0.001 |
| 11 | Benzilan, YN | 28°15′N | 99°16′W | 2487 | 7 |  | 7 |  |  |  |  |  |  |  |  |  |  |  |  |  |  | 0.000 | 0.000 |
| 12 | Baima Mt, YN | 28°18′N | 99°08′W | 3443 | 5 |  | 5 |  |  |  |  |  |  |  |  |  |  |  |  |  |  | 0.000 | 0.000 |
| 13 | Wujing, YN | 27°38′N | 99°29′W | 1904 | 5 |  | 4 |  |  | 1 |  |  |  |  |  |  |  |  |  |  |  | 0.400 | 0.001 |
| 14 | Lanping, YN | 25°50′N | 98°51′W | 824 | 16 |  |  | 7 | 9 |  |  |  |  |  |  |  |  |  |  |  |  | 0.525 | 0.000 |
| P. | Location | Latitude | Longitude | Alt.(m) | N | H1 | H2 | H3 | H4 | H5 | H6 | H7 | H8 | H9 | H10 | H11 | H12 | H13 | H14 | H15 | H16 | *H_e_* | *π_e_* |
| *(continued)* | | | | | | | | | | | | | | | | | | | | | | | |
| 15 | Yulong, YN | 26°45′N | 99°56′W | 2408 | 5 |  |  | 5 |  |  |  |  |  |  |  |  |  |  |  |  |  | 0.000 | 0.000 |
| 16 | Eryuan, YN | 26°06′N | 99°55′W | 2057 | 4 |  |  | 4 |  |  |  |  |  |  |  |  |  |  |  |  |  | 0.000 | 0.000 |
| 17 | Yunlong, YN | 26°04′N | 99°07′W | 1359 | 5 |  |  | 5 |  |  |  |  |  |  |  |  |  |  |  |  |  | 0.000 | 0.000 |
| 18 | Shigu, YN | 26°52′N | 99°59′W | 1825 | 15 |  |  | 15 |  |  |  |  |  |  |  |  |  |  |  |  |  | 0.000 | 0.000 |
| 19 | Lijiang, YN | 26°57′N | 100°12′W | 2657 | 9 |  |  | 6 |  |  | 3 |  |  |  |  |  |  |  |  |  |  | 0.500 | 0.001 |
| 20 | Hutiaoxia, YN | 27°13′N | 100°07′W | 2587 | 5 |  |  | 5 |  |  |  |  |  |  |  |  |  |  |  |  |  | 0.000 | 0.000 |
| 21 | Jianchuan, YN | 26°25′N | 99°59′W | 2534 | 4 |  |  | 1 | 2 |  |  | 1 |  |  |  |  |  |  |  |  |  | 0.833 | 0.001 |
| 22 | Heqing, YN | 26°20′N | 100°12′W | 2020 | 14 |  |  | 3 |  |  | 5 | 5 | 1 |  |  |  |  |  |  |  |  | 0.747 | 0.001 |
| 23 | Dali, YN | 26°10′N | 100°09′W | 2539 | 9 |  |  | 5 | 4 |  |  |  |  |  |  |  |  |  |  |  |  | 0.556 | 0.001 |
| 24 | Nilang, YN | 27°19′N | 100°51′W | 2243 | 12 |  |  | 10 |  |  | 1 | 1 |  |  |  |  |  |  |  |  |  | 0.318 | 0.000 |
| 25 | Muli, SC | 27°44′N | 101°13′W | 2852 | 18 |  |  | 7 |  |  | 3 | 3 |  |  |  |  |  |  |  |  | 5 | 0.758 | 0.002 |
| 26 | Yanyuan, SC | 27°25′N | 101°30′W | 2540 | 6 |  |  | 1 |  |  |  | 5 |  |  |  |  |  |  |  |  |  | 0.333 | 0.000 |
| 27 | Derong, SC | 28°51′N | 99°18′W | 2594 | 13 |  | 2 | 11 |  |  |  |  |  |  |  |  |  |  |  |  |  | 0.282 | 0.000 |
| 28 | Xiangcheng, SC | 29°00′N | 99°45′W | 3471 | 37 | 6 | 8 |  |  |  | 23 |  |  |  |  |  |  |  |  |  |  | 0.556 | 0.001 |
| 29 | Litang, SC | 30°31′N | 100°19′W | 2859 | 15 | 15 |  |  |  |  |  |  |  |  |  |  |  |  |  |  |  | 0.000 | 0.000 |
| 30 | Jiulong, SC | 29°36′N | 101°21′W | 3136 | 28 |  |  | 27 |  |  |  |  | 1 |  |  |  |  |  |  |  |  | 0.071 | 0.000 |
| P. | Location | Latitude | Longitude | Alt.(m) | N | H1 | H2 | H3 | H4 | H5 | H6 | H7 | H8 | H9 | H10 | H11 | H12 | H13 | H14 | H15 | H16 | *H_e_* | *π_e_* |
| *(continued)* | | | | | | | | | | | | | | | | | | | | | | | |
| 31 | Yajiang, SC | 30°02′N | 100°59W | 2627 | 7 |  |  | 5 |  |  |  |  | 2 |  |  |  |  |  |  |  |  | 0.476 | 0.001 |
| 32 | Daofu, SC | 30°49′N | 101°16′W | 4100 | 7 |  |  |  |  |  |  |  | 7 |  |  |  |  |  |  |  |  | 0.000 | 0.000 |
| 33 | Luding, SC | 30°06′N | 102°10′W | 1381 | 55 |  |  | 2 |  |  |  |  | 51 |  |  |  |  | 2 |  |  |  | 0.140 | 0.000 |
| 34 | Danba, SC | 31°52′N | 101°59′W | 2369 | 12 |  |  |  |  |  |  |  | 10 |  |  |  |  |  | 1 | 1 |  | 0.318 | 0.000 |
| 35 | Jinchuan, SC | 31°36′N | 102°04′W | 2157 | 23 |  |  |  |  |  |  |  | 23 |  |  |  |  |  |  |  |  | 0.000 | 0.000 |
| 36 | Maerkang, SC | 31°55′N | 102°11′W | 2602 | 10 |  |  |  |  |  |  |  | 10 |  |  |  |  |  |  |  |  | 0.000 | 0.000 |
| 37 | Heishui, SC | 32°08′N | 103°09′W | 2350 | 8 |  |  |  |  |  |  |  | 8 |  |  |  |  |  |  |  |  | 0.000 | 0.000 |
| 38 | Maoxian, SC | 31°32′N | 103°41′W | 1462 | 15 |  |  |  |  |  |  |  | 15 |  |  |  |  |  |  |  |  | 0.000 | 0.000 |
|  | All populations | | | | 477 |  |  |  |  |  |  |  |  |  |  |  |  |  |  |  |  | 0.308 | 0.001 |
|  |  | | | |  |  |  |  |  |  |  |  |  |  |  |  |  |  |  |  |  |  |  |

Abbreviations: P., the population code; Alt.,altitude; TB, Tibet; YN, Yunnan; SC, Sichuan; N, sample size.

**Table S2**. Variable sites of all sixteen *mat*K haplotypes observed in *O. sinensis*. The length of aligned sequences is 1173 bp. The most common haplotype H3 was used as a reference and matchingnucleotides in others were denoted by “.”. The haplotype sequences are available in Genbank database under accession number KJ159010 - KJ159025.

|  | 39 | 51 | 115 | 156 | 274 | 304 | 477 | 509 | 660 | 668 | 681 | 691 | 777 | 789 | 842 | 950 | 1003 | 1154 |
| --- | --- | --- | --- | --- | --- | --- | --- | --- | --- | --- | --- | --- | --- | --- | --- | --- | --- | --- |
| *Phylogenetic clade A* | | | | | | | | | | | | | | | | | | |
| H3 | C | T | C | C | C | G | C | G | C | A | C | G | T | C | G | C | C | C |
| H1 | . | . | . | A | . | . | . | . | . | . | . | . | . | . | . | . | . | . |
| H2 | . | . | . | . | . | . | . | . | . | . | . | A | . | . | . | . | . | . |
| H4 | . | G | . | . | . | . | . | . | . | . | . | . | . | . | . | . | . | . |
| H5 | . | . | . | . | . | . | . | . | T | . | . | . | . | . | . | . | . | . |
| H7 | . | . | . | . | . | . | . | . | . | . | . | . | . | . | . | T | . | . |
| H11 | . | . | . | . | . | . | . | . | . | . | . | . | . | A | . | . | . | . |
| H16 | . | . | . | . | A | . | . | . | . | T | . | . | . | . | . | . | . | . |
| *Phylogenetic clade B* | | | | | | | | | | | | | | | | | | |
| H6 | A | . | . | . | . | . | . | . | . | . | A | . | . | . | . | . | . | . |
| H8 | . | . | . | . | . | . | . | . | . | . | A | . | . | . | A | . | . | . |
| H13 | . | . | . | . | . | . | . | . | . | . | A | . | . | . | A | . | . | T |
| H14 | . | . | . | . | . | . | . | . | . | T | A | . | . | . | A | . | . | . |
| H15 | . | . | . | . | . | . | . | . | . | T | A | . | . | . | A | . | . | T |
| *Phylogenetic clade C* | | | | | | | | | | | | | | | | | | |
| H9 | . | . | A | . | . | T | . | T | . | . | A | . | C | . | . | . | . | . |
| H10 | . | . | A | . | . | T | A | T | . | . | A | . | C | . | . | . | . | . |
| H12 | . | . | A | . | . | T | A | T | . | . | A | . | C | . | . | . | T | . |

**Table S3**. Specimen records of *O. sinensis* in the [Chinese Virtual Herbarium](http://www.cvh.ac.cn/news/8) (CNV). Abbreviation: HNWP = Northwest Institute of Plateau Biology,Chinese Academy of Sciences; PE = Institute of Botany, Chinese Academy of Sciences; KUN = Kunming Institute of Botany, Chinese Academy of Sciences; IBSC = South China Botanical Garden, Chinese Academy of Sciences; WUK = Northwest Agriculture & Forestry University; HITBC = Xishuangbanna Tropical Botanical Garden, Chinese Academy of Sciences.

| **Species** | **Herbarium/ID** | **Latitude** | **Longitude** | **Location** | **Collect Date** |
| --- | --- | --- | --- | --- | --- |
| ***O. sinensis*** | HNWP/65649 | 30.98 | 101.13 | Daofu, Sichuan | 19770705 |
| ***O. sinensis*** | PE/00240086 | 32.05 | 102.58 | Heishui, Sichuan | 19590507 |
| ***O. sinensis*** | PE/00513466 | 31.46 | 102.04 | Jinchuan, Sichuan | 19830615 |
| ***O. sinensis*** | PE/00513467 | 31.46 | 102.04 | Jinchuan, Sichuan | 19830615 |
| ***O. sinensis*** | PE/00513468 | 31.46 | 102.04 | Jinchuan, Sichuan | 19830615 |
| ***O. sinensis*** | KUN/0122919 | 29.00 | 101.51 | Jiulong, Sichuan | 19300000 |
| ***O. sinensis*** | IBSC/0152212 | 29.00 | 101.51 | Jiulong, Sichuan | 19790630 |
| ***O. sinensis*** | KUN/0122914 | 31.91 | 102.21 | Maerkang, Sichuan | 19570509 |
| ***O. sinensis*** | KUN/0122907 | 27.90 | 102.27 | Xichang, Sichuan | 19760426 |
| ***O. sinensis*** | PE/00240138 | 28.24 | 102.51 | Yuexi, Sichuan | 19320603 |
| ***O. sinensis*** | WUK/307882 | 33.01 | 99.13 | Batang, Sichuan | 197507 |
| ***O. sinensis*** | PE/00240108 | 30.03 | 96.93 | Basu, Tibet | 19760704 |
| ***O. sinensis*** | PE/00240132 | 29.68 | 98.62 | Mangkang, Tibet | 19760601 |
| ***O. sinensis*** | PE/00240111 | 29.68 | 98.62 | Mangkang, Tibet, | 19760603 |
| ***O. sinensis*** | KUN/0122883 | 28.49 | 98.91 | Deqin, Yunnan | 19370525 |
| ***O. sinensis*** | KUN/0122903 | 27.74 | 98.67 | Gongshan, Yunnan | 19780714 |
| ***O. sinensis*** | KUN/0122904 | 27.74 | 98.67 | Gongshan, Yunnan | 19780715 |
| ***O. sinensis*** | KUN/0426321 | 26.88 | 100.23 | Lijiang, Yunnan | 19850604 |
| ***O. sinensis*** | KUN/0122895 | 26.88 | 100.23 | Lijiang, Yunnan | 19620508 |
| ***O. sinensis*** | KUN/0122896 | 26.88 | 100.23 | Lijiang, Yunnan | 19620509 |
| ***O. sinensis*** | PE/00240135 | 28.87 | 107.61 | Daozhen, Guizhou | 19960720 |
| ***O. sinensis*** | PE/00240077 | 33.00 | 99.12 | Batang,Sichuan | 19860506 |
| ***O. sinensis*** | PE/00240141 | 30.36 | 102.82 | Baoxing, Sichuan | 19590427 |
| ***O. sinensis*** | WUK/253548 | 31.46 | 102.09 | Dajin, Sichuan | 195804 |
| ***O. sinensis*** | PE/00240136 | 31.44 | 102.10 | Dajin, Sichuan | 19610515 |
| ***O. sinensis*** | PE/00240137 | 31.44 | 102.10 | Dajin, Sichuan | 19610515 |
| ***O. sinensis*** | PE/00240061 | 31.46 | 102.09 | Dajin, Sichuan, | 19580404 |
| ***O. sinensis*** | PE/00240064 | 31.46 | 102.09 | Dajin, Sichuan, | 19580404 |
| ***O. sinensis*** | PE/00240069 | 31.46 | 102.09 | Dajin, Sichuan, | 19580404 |
| ***O. sinensis*** | PE/00240093 | 30.98 | 101.11 | Daofu, Sichuan | 19510919 |
| ***O. sinensis*** | PE/00240084 | 29.04 | 100.30 | Daocheng, Sichuan | 19730707 |
| ***O. sinensis*** | KUN/0122918 | 29.04 | 100.30 | Daocheng, Sichuan | 19730707 |
| ***O. sinensis*** | PE/00240085 | 31.79 | 98.63 | Dege, Sichuan | 19740713 |
| ***O. sinensis*** | PE/00240143 | 29.58 | 103.54 | Emei, Sichuan | 19950425 |
| ***O. sinensis*** | PE/00240073 | 32.06 | 102.99 | Heishui, Sichuan | 19590507 |
| ***O. sinensis*** | KUN/0122913 | 32.06 | 102.99 | Heishui, Sichuan | 19590507 |
| ***O. sinensis*** | KUN/0122921 | 26.63 | 102.58 | Huidong, Sichuan | 19590628 |
| ***O. sinensis*** | PE/00497680 | 31.46 | 102.04 | Jinchuan, Sichuan | 19830615 |
| ***O. sinensis*** | IBSC/0152210 | 29.00 | 101.51 | Jiulong, Sichuan | 19800615 |
| ***O. sinensis*** | WUK/303182 | 30.06 | 101.96 | Kangding, Sichuan | 197404 |
| ***O. sinensis*** | PE/00240075 | 30.04 | 101.98 | Kangding, Sichuan | 19610523 |
| ***O. sinensis*** | PE/00240076 | 30.09 | 102.01 | Kangding, Sichuan | 19860429 |
| ***O. sinensis*** | PE/00240067 | 30.05 | 101.98 | Kangding, Sichuan | 19740419 |
| ***O. sinensis*** | PE/00240071 | 30.10 | 102.03 | Kangding, Sichuan | 19520421 |
| ***O. sinensis*** | PE/00240079 | 30.05 | 101.98 | Kangding, Sichuan | 19600515 |
| ***O. sinensis*** | PE/00240080 | 30.09 | 101.99 | Kangding, Sichuan | 19630715 |
| ***O. sinensis*** | PE/00240081 | 30.09 | 101.99 | Kangding, Sichuan | 19630715 |
| ***O. sinensis*** | PE/00240082 | 30.09 | 101.99 | Kangding, Sichuan | 19630715 |
| ***O. sinensis*** | PE/00240096 | 30.12 | 101.95 | Kangding, Sichuan | 19740524 |
| ***O. sinensis*** | KUN/0122917 | 30.06 | 101.96 | Kangding, Sichuan | 19590624 |
| ***O. sinensis*** | WUK/252631 | 31.91 | 102.21 | Maerkang, Sichuan | 195706 |
| ***O. sinensis*** | WUK/242701 | 31.91 | 102.21 | Maerkang, Sichuan | 195707 |
| ***O. sinensis*** | WUK/252336 | 31.91 | 102.21 | Maerkang, Sichuan | 195707 |
| ***O. sinensis*** | WUK/251736 | 31.91 | 102.21 | Maerkang, Sichuan | 195705 |
| ***O. sinensis*** | WUK/252363 | 31.91 | 102.21 | Maerkang, Sichuan | 195705 |
| ***O. sinensis*** | KUN/0122922 | 31.91 | 102.21 | Maerkang, Sichuan | 19300612 |
| ***O. sinensis*** | PE/00240063 | 31.91 | 102.21 | Maerkang, Sichuan | 19570413 |
| ***O. sinensis*** | PE/00240068 | 31.91 | 102.21 | Maerkang, Sichuan | 19570508 |
| ***O. sinensis*** | PE/00240088 | 31.91 | 102.21 | Maerkang, Sichuan | 19570604 |
| ***O. sinensis*** | PE/00240089 | 31.91 | 102.21 | Maerkang, Sichuan | 19570715 |
| ***O. sinensis*** | PE/00240090 | 31.91 | 102.21 | Maerkang, Sichuan | 19570517 |
| ***O. sinensis*** | PE/00240095 | 31.91 | 102.21 | Maerkang, Sichuan | 19570509 |
| ***O. sinensis*** | PE/00240098 | 31.91 | 102.21 | Maerkang, Sichuan | 19570423 |
| ***O. sinensis*** | PE/00240099 | 31.91 | 102.21 | Maerkang, Sichuan | 19570416 |
| ***O. sinensis*** | PE/00240100 | 31.91 | 102.21 | Maerkang, Sichuan | 19570516 |
| ***O. sinensis*** | IBSC/0152200 | 31.91 | 102.21 | Maerkang, Sichuan | 19570416 |
| ***O. sinensis*** | KUN/0122920 | 31.91 | 102.21 | Maerkang, Sichuan | 19570715 |
| ***O. sinensis*** | KUN/0122916 | 31.91 | 102.21 | Maerkang, Sichuan | 19570604 |
| ***O. sinensis*** | IBSC/0152208 | 31.91 | 102.21 | Maerkang, Sichuan | 19570508 |
| ***O. sinensis*** | IBSC/0152204 | 31.91 | 102.21 | Maerkang, Sichuan | 19570516 |
| ***O. sinensis*** | KUN/0122915 | 31.91 | 102.21 | Maerkang, Sichuan | 19570516 |
| ***O. sinensis*** | IBSC/0152206 | 31.91 | 102.21 | Maerkang, Sichuan | 19570509 |
| ***O. sinensis*** | IBSC/0152207 | 31.91 | 102.21 | Maerkang, Sichuan | 19570423 |
| ***O. sinensis*** | IBSC/0152203 | 31.91 | 102.21 | Maerkang, Sichuan | 19570604 |
| ***O. sinensis*** | PE/00240091 | 31.68 | 103.85 | Maowen, Sichuan | 19590608 |
| ***O. sinensis*** | PE/00240094 | 31.68 | 103.85 | Maowen, Sichuan | 19590826 |
| ***O. sinensis*** | PE/00240074 | 28.54 | 102.22 | Mianning, Sichuan | 19760609 |
| ***O. sinensis*** | PE/00513469 | 27.97 | 101.20 | Muli, Sichuan | 19820605 |
| ***O. sinensis*** | PE/00513470 | 27.97 | 101.20 | Muli, Sichuan | 19820605 |
| ***O. sinensis*** | PE/00513471 | 27.97 | 101.20 | Muli, Sichuan | 19820605 |
| ***O. sinensis*** | PE/00240101 | 27.97 | 101.20 | Muli, Sichuan, | 19370527 |
| ***O. sinensis*** | PE/00240102 | 27.93 | 101.28 | Muli, Sichuan, | 19370610 |
| ***O. sinensis*** | PE/00240103 | 27.93 | 101.28 | Muli, Sichuan, | 19370610 |
| ***O. sinensis*** | KUN/0122910 | 27.93 | 101.28 | Muli, Sichuan | 19370610 |
| ***O. sinensis*** | KUN/0122911 | 27.93 | 101.28 | Muli, Sichuan | 19370610 |
| ***O. sinensis*** | PE/00240134 | 29.16 | 107.11 | Nanchuan, Sichuan | 19960511 |
| ***O. sinensis*** | WUK/283335 | 29.23 | 102.36 | Shimian, Sichuan | 1955 |
| ***O. sinensis*** | WUK/175810 | 29.23 | 102.36 | Shimian, Sichuan | 195504 |
| ***O. sinensis*** | PE/00240078 | 29.23 | 102.36 | Shimian, Sichuan | 19550000 |
| ***O. sinensis*** | IBSC/0152201 | 29.23 | 102.36 | Shimian, Sichuan | 19550000 |
| ***O. sinensis*** | PE/00240070 | 31.08 | 103.27 | Wenchuan, Sichuan | 19520421 |
| ***O. sinensis*** | IBSC/0152199 | 31.08 | 103.27 | Wenchuan, Sichuan | 19520431 |
| ***O. sinensis*** | WUK/303679 | 28.93 | 99.80 | Xiangcheng, Sichuan | 197404 |
| ***O. sinensis*** | PE/00240097 | 28.93 | 99.80 | Xiangcheng, Sichuan | 19740423 |
| ***O. sinensis*** | PE/00240083 | 28.93 | 99.80 | Xiangcheng, Sichuan, | 19720617 |
| ***O. sinensis*** | PE/00240142 | 31.00 | 102.39 | Xiaojin, Sichuan | 19300612 |
| ***O. sinensis*** | PE/00240092 | 31.00 | 102.39 | Xiaojin, Sichuan | 19570509 |
| ***O. sinensis*** | PE/00240065 | 30.02 | 101.08 | Yajiang, Sichuan | 19600410 |
| ***O. sinensis*** | KUN/0122912 | 27.42 | 101.51 | Yanyuan, Sichuan | 19600424 |
| ***O. sinensis*** | PE/00240072 | 27.42 | 101.51 | Yanyuan, Sichuan | 19600424 |
| ***O. sinensis*** | PE/00240140 | 28.24 | 102.51 | Yuexi, Sichuan | 19320000 |
| ***O. sinensis*** | KUN/0122909 | 28.01 | 102.84 | Zhaojue, Sichuan | 19851025 |
| ***O. sinensis*** | WUK/48017 | 29.72 | 99.22 | Derong, Sichuan | 193006 |
| ***O. sinensis*** | WUK/307881 | 33.01 | 99.13 | Batang, Sichuan | 197507 |
| ***O. sinensis*** | IBSC/0152211 | 32.26 | 100.97 | zhuosijia, Sichuan | 19580614 |
| ***O. sinensis*** | HITBC/073072 | 28.76 | 99.29 | Derong, Sichuan | 19810702 |
| ***O. sinensis*** | HITBC/006259 | 28.76 | 99.29 | Derong, Sichuan | 19810702 |
| ***O. sinensis*** | HITBC/073067 | 27.93 | 101.28 | Muli, Sichuan | 19830825 |
| ***O. sinensis*** | KUN/0122908 | 31.91 | 102.21 | Xikang, Sichuan | 19410000 |
| ***O. sinensis*** | PE/00240133 | 30.03 | 96.93 | Basu, Tibet | 19760704 |
| ***O. sinensis*** | PE/00046502 | 30.03 | 96.93 | Basu, Tibet | 19760508 |
| ***O. sinensis*** | HNWP/61155 | 30.05 | 96.92 | Basu, Tibet | 19760704 |
| ***O. sinensis*** | KUN/0122929 | 30.05 | 96.92 | Basu, Tibet | 19760704 |
| ***O. sinensis*** | KUN/0122930 | 30.05 | 96.92 | Basu, Tibet | 19760704 |
| ***O. sinensis*** | PE/00240107 | 28.67 | 97.47 | Chayu, Tibet | 19800629 |
| ***O. sinensis*** | PE/00497679 | 28.67 | 97.47 | Chayu, Tibet | 19730808 |
| ***O. sinensis*** | PE/00240104 | 28.67 | 97.47 | Chayu, Tibet, | 19800629 |
| ***O. sinensis*** | PE/00240105 | 28.67 | 97.47 | Chayu, Tibet, | 19800813 |
| ***O. sinensis*** | KUN/0122924 | 29.14 | 92.59 | Jiacha, Tibet | 19750710 |
| ***O. sinensis*** | PE/00240131 | 29.68 | 98.62 | Mangkang, Tibet | 19760603 |
| ***O. sinensis*** | PE/00240110 | 29.68 | 98.62 | Mangkang, Tibet, | 19760601 |
| ***O. sinensis*** | PE/00046497 | 29.68 | 98.62 | Mangkang, Tibet | 19760601 |
| ***O. sinensis*** | PE/00046847 | 29.68 | 98.62 | Mangkang, Tibet | 19760601 |
| ***O. sinensis*** | HNWP/61775 | 29.68 | 98.59 | Mangkang, Tibet | 19760601 |
| ***O. sinensis*** | KUN/0122925 | 29.68 | 98.59 | Mangkang, Tibet | 19760601 |
| ***O. sinensis*** | KUN/0122926 | 29.68 | 98.59 | Mangkang, Tibet | 19760601 |
| ***O. sinensis*** | KUN/0122927 | 29.68 | 98.59 | Mangkang, Tibet | 19760603 |
| ***O. sinensis*** | KUN/0122928 | 29.68 | 98.59 | Mangkang, Tibet | 19760603 |
| ***O. sinensis*** | HNWP/61730 | 29.68 | 98.59 | Mangkang, Tibet | 19760603 |
| ***O. sinensis*** | WUK/344353 | 31.46 | 98.23 | Changdu, Tibet | 197707 |
| ***O. sinensis*** | PE/00240106 | 31.66 | 98.66 | Nujiang, Tibet | 19860509 |
| ***O. sinensis*** | PE/00240109 | 31.66 | 98.66 | Nujiang, Tibet | 19650706 |
| ***O. sinensis*** | PE/00240130 | 31.91 | 102.21 | Xikangyanduo, Tibet | 19510919 |
| ***O. sinensis*** | KUN/0122876 | 25.58 | 100.22 | Dali, Yunnan | 19450500 |
| ***O. sinensis*** | KUN/0122871 | 25.58 | 100.22 | Dali, Yunnan | 19410800 |
| ***O. sinensis*** | KUN/0122872 | 25.58 | 100.22 | Dali, Yunnan | 19410800 |
| ***O. sinensis*** | KUN/0122873 | 25.58 | 100.22 | Dali, Yunnan | 19420803 |
| ***O. sinensis*** | KUN/0122874 | 25.58 | 100.22 | Dali, Yunnan | 19450326 |
| ***O. sinensis*** | KUN/0122875 | 25.58 | 100.22 | Dali, Yunnan | 19450326 |
| ***O. sinensis*** | PE/00240119 | 28.49 | 98.91 | Deqin, Yunnan | 19370525 |
| ***O. sinensis*** | PE/00240123 | 28.49 | 98.91 | Deqin, Yunnan | 19370525 |
| ***O. sinensis*** | KUN/0122877 | 28.49 | 98.91 | Deqin, Yunnan | 19810705 |
| ***O. sinensis*** | KUN/0122878 | 28.49 | 98.91 | Deqin, Yunnan | 19810705 |
| ***O. sinensis*** | KUN/0122884 | 28.49 | 98.91 | Deqin, Yunnan | 19370525 |
| ***O. sinensis*** | PE/00240114 | 26.17 | 103.30 | Dongchuan, Yunnan | 19850503 |
| ***O. sinensis*** | PE/00240115 | 26.17 | 103.30 | Dongchuan, Yunnan | 19850503 |
| ***O. sinensis*** | PE/00240128 | 26.17 | 103.30 | Dongchuan, Yunnan | 19850503 |
| ***O. sinensis*** | KUN/0122889 | 27.74 | 98.67 | Gongshan, Yunnan | 19600529 |
| ***O. sinensis*** | KUN/0122890 | 27.74 | 98.67 | Gongshan, Yunnan | 19380905 |
| ***O. sinensis*** | KUN/0122891 | 26.42 | 103.30 | Huize, Yunnan | 19950601 |
| ***O. sinensis*** | IBSC/0152215 | 26.88 | 100.23 | Lijiang, Yunnan | 19060500 |
| ***O. sinensis*** | PE/00240118 | 26.88 | 100.23 | Lijiang, Yunnan | 19350600 |
| ***O. sinensis*** | PE/00240127 | 26.88 | 100.23 | Lijiang, Yunnan | 19400509 |
| ***O. sinensis*** | PE/00240129 | 26.88 | 100.23 | Lijiang, Yunnan | 19400509 |
| ***O. sinensis*** | HITBC/006260 | 26.88 | 100.23 | Lijiang, Yunnan | 19810530 |
| ***O. sinensis*** | KUN/0122886 | 26.88 | 100.23 | Lijiang, Yunnan | 19580831 |
| ***O. sinensis*** | KUN/0426320 | 26.88 | 100.23 | Lijiang, Yunnan | 19850604 |
| ***O. sinensis*** | KUN/0122892 | 26.88 | 100.23 | Lijiang, Yunnan | 19810512 |
| ***O. sinensis*** | KUN/0122901 | 26.88 | 100.23 | Lijiang, Yunnan | 19400509 |
| ***O. sinensis*** | KUN/0122902 | 26.88 | 100.23 | Lijiang, Yunnan | 19400509 |
| ***O. sinensis*** | KUN/0122893 | 26.88 | 100.23 | Lijiang, Yunnan | 19620508 |
| ***O. sinensis*** | KUN/0122894 | 26.88 | 100.23 | Lijiang, Yunnan | 19620508 |
| ***O. sinensis*** | KUN/0122899 | 26.88 | 100.23 | Lijiang, Yunnan | 19600420 |
| ***O. sinensis*** | KUN/0122900 | 26.88 | 100.23 | Lijiang, Yunnan | 19600603 |
| ***O. sinensis*** | KUN/0122881 | 27.28 | 100.85 | Ninglang, Yunnan | 19370501 |
| ***O. sinensis*** | KUN/0122882 | 27.28 | 100.85 | Ninglang, Yunnan | 19370501 |
| ***O. sinensis*** | PE/00240124 | 26.89 | 102.98 | Qiaojia, Yunnan | 19960805 |
| ***O. sinensis*** | PE/00240117 | 27.18 | 99.29 | Weixi, Yunnan | 19350800 |
| ***O. sinensis*** | PE/00240125 | 27.18 | 99.29 | Weixi, Yunnan | 19400603 |
| ***O. sinensis*** | PE/00240126 | 27.18 | 99.29 | Weixi, Yunnan | 19400603 |
| ***O. sinensis*** | KUN/0122897 | 27.18 | 99.29 | Weixi, Yunnan | 19400603 |
| ***O. sinensis*** | KUN/0122898 | 27.18 | 99.29 | Weixi, Yunnan | 19400603 |
| ***O. sinensis*** | KUN/0122885 | 27.18 | 99.29 | Weixi, Yunnan | 19350800 |
| ***O. sinensis*** | PE/00240112 | 27.29 | 100.96 | Yongning, Yunnan | 19370501 |
| ***O. sinensis*** | PE/00240113 | 27.29 | 100.96 | Yongning, Yunnan | 19370501 |
| ***O. sinensis*** | PE/00240121 | 25.88 | 99.38 | Yunlong, Yunnan | 19430320 |
| ***O. sinensis*** | PE/00240116 | 27.34 | 103.71 | Zhaotong, Yunnan | 19320505 |
| ***O. sinensis*** | PE/00240122 | 27.34 | 103.71 | Zhaotong, Yunnan | 19320505 |
| ***O. sinensis*** | KUN/0122879 | 27.34 | 103.71 | Zhaotong, Yunnan | 19320505 |
| ***O. sinensis*** | KUN/0122880 | 27.34 | 103.71 | Zhaotong, Yunnan | 19320505 |
| ***O. sinensis*** | HITBC/073071 | 28.49 | 98.91 | Deqin, Yunnan | 1981 |
| ***O. sinensis*** | HITBC/076717 | 26.88 | 100.23 | Lijiang, Yunnan | 19810000 |
| ***O. sinensis*** | HITBC/006257 | 28.35 | 99.19 | Zhongdian, Yunnan | 19810629 |
| ***O. sinensis*** | HITBC/006258 | 28.35 | 99.19 | Zhongdian, Yunnan | 19810705 |
| ***O. sinensis*** | IBSC/0152209 | 31.91 | 102.21 | Maerkang, Sichuan | 19570715 |


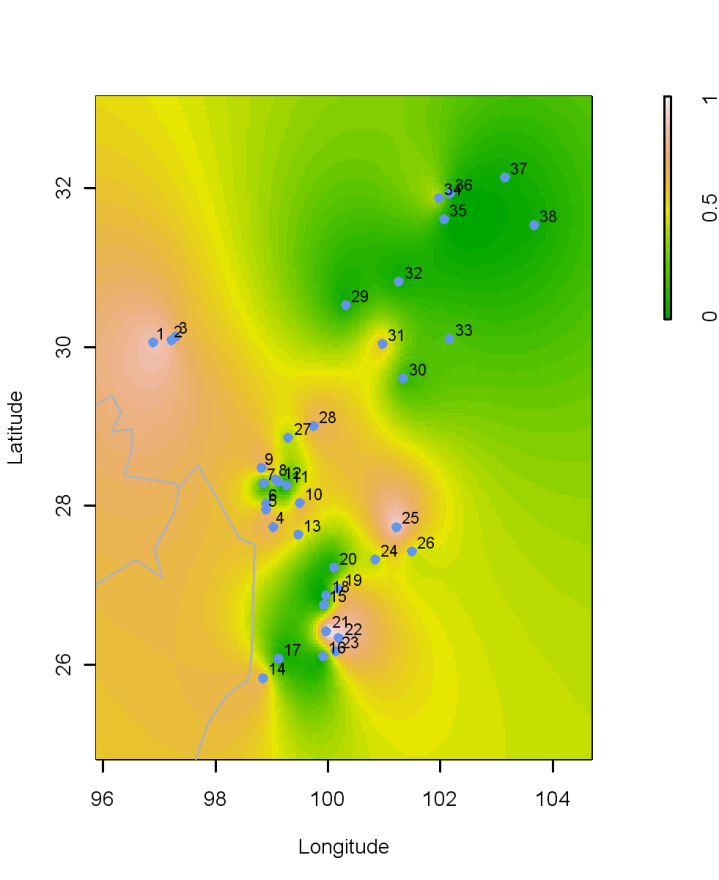


**Figure S1.** The interpolated spatial distribution of haplotype diversity of *O.sinensis*. The blue points depict the sampling locations. The kriging surface was evaluated over a grid under Gaussian random spatial processes in the R “spatial” package. The map was plotted using a modified script from “spatial” packages in the R statistic environment.


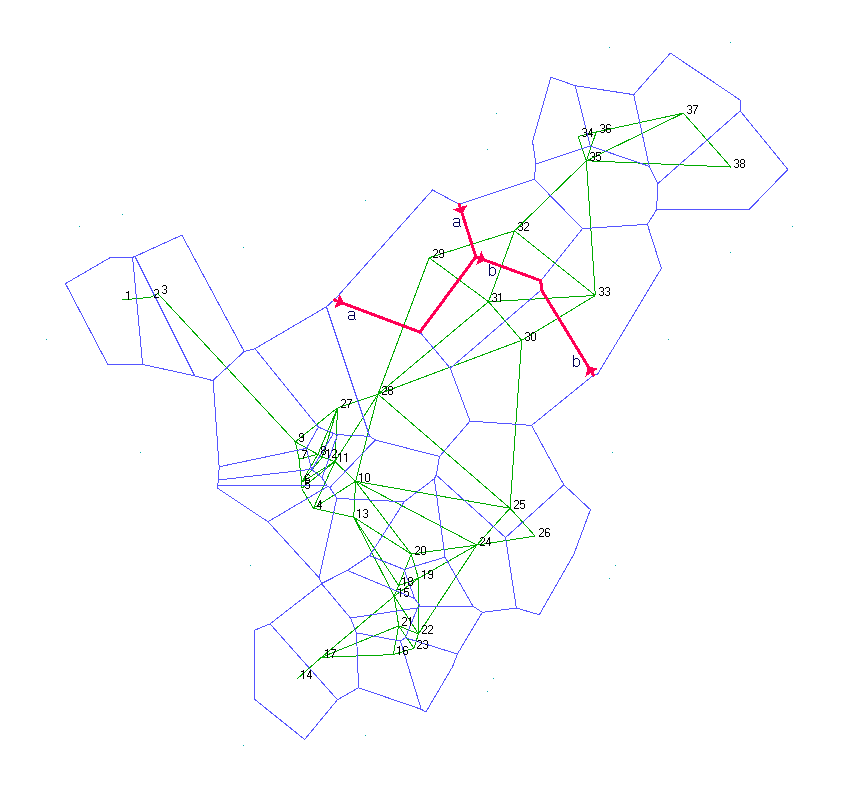


**Figure S2.** Results of the BARRIER analysis based on pairwise Fst values of all populations, showing the spatial separation of O. sinensis. Delaunay triangulation was shown as thin blue connecting lines, and the primary and secondary barriers (thick red line) separated all populations into three groups with main discontinuity occurred between Himalaya-Hengduan Mountains regions and east QTP.
